# Deep neural networks in medical microbiology for bacterial colonies classification

**DOI:** 10.1038/s41598-026-48621-0

**Published:** 2026-05-12

**Authors:** José Duarte Pereira, Bruno Veloso, João Gama

**Affiliations:** 1 Faculty of Economics, University of Porto, Rua Dr. Roberto Frias, Porto, 4200-464 Portugal; 2 Clinical Pathology Service, Portuguese Oncology Institute of Porto, Rua Dr. António Bernardino de Almeida, 4200-072 Porto, Portugal; 3 Institute for Systems and Computer Engineering, Technology and Science (INESC TEC), Rua Dr. Roberto Frias, 4200-465 Porto, Portugal

**Keywords:** Biological techniques, Computational biology and bioinformatics, Microbiology

## Abstract

While automation has transformed many areas inside clinical laboratories, microbiology still relies heavily on manual tasks, particularly the culture of samples on agar plates and their subsequent manual review for microorganism identification and antibiotic susceptibility profiling. Bacterial colony detection and classification require trained professionals, making the process time-consuming and prone to human error. Developing deep learning models to automate these tasks could improve microbiology workflows and accelerate clinical decision-making. In this study we trained and evaluated five object detection architectures (Faster R-CNN and RetinaNet with ResNet-50 and ResNet-101 backbones, and YOLOv8) on the Annotated Germs for Automated Recognition (AGAR) dataset for bacterial colony classification. Transfer learning, cross-subset generalization, and Weighted Box Fusion (WBF) ensemble methods were applied to enhance and characterize performance. Additionally, we created and publicly released a curated dataset of 165 agar plate images containing colonies of *S. aureus*, *P. aeruginosa*, and *E. coli* cultured across four distinct culture media. YOLOv8m achieved a mean Average Precision (mAP) of 69.0% on the AGAR dataset, outperforming the best Detectron2 model (Faster R-CNN ResNet-101, 63.1%) by 5.9 percentage points. A four-model WBF ensemble combining both architectures reached 70.5% mAP (95% CI: 68.4–71.7). Cross-subset evaluation showed that a single model trained on the full dataset generalizes well to individual imaging conditions, making subset-specific fine-tuning largely unnecessary. On the curated dataset, a mixed ensemble reached 58.7% mAP (95% CI: 57.1–63.7). These results demonstrate that architecture choice and training data diversity are the primary drivers of performance for colony detection on agar plates.

## Introduction

Advancements in medical laboratory technology have enabled the integration of expert systems and software applications, such as automated analyzers and laboratory information system modules. This is evident in haematology and clinical chemistry, where automation is currently part of daily routines^1–4^. However, the microbiology sector still relies heavily on manual, time-intensive processes. Tasks such as sample preparation, microscopy slides visualization, culturing samples on agar plates, analyzing microbial colonies, and antimicrobial profile testing are labour and time-intensive and delay medical reports, impacting patient care. The manual nature of these workflows can also limit operational efficiency, creating a demand for automated solutions that improve standardization, efficiency and safety, reduce resource strain and enhance long-term cost-effectiveness^1,5,6^.

Artificial intelligence (AI) has made significant strides in medical imaging, mainly through machine learning (ML) and deep learning (DL). DL methods, especially convolutional neural networks (CNNs), have demonstrated high accuracy in radiology^7^, histology^8^, and haematology^9^, where image-based data plays a central role. Since laboratories are primary suppliers of quantitative, structured, and codified data^6^, these advancements highlight the significant potential of ML and DL to create and enhance automated tools within laboratory settings, extending naturally to microbiology.

While studies have investigated DL applications in microbiology (colony classification on chromogenic media, detection of mycobacteria in sputum samples, identification of malaria parasites in blood smears)^5,10^, this field still lacks automated solutions that can directly and accurately detect and classify bacterial colonies on agar plates for clinical use. This is compounded by the diversity of sample types, processing techniques, and cost concerns^1,5^.

Nevertheless, ongoing research in DL has produced promising results for addressing these challenges. The evolution of these technologies for the detection and classification of microbial colonies would tackle a time-consuming and error-prone process in microbiology labs. Automated image analysis algorithms could execute computer-assisted culture interpretation, enabling the rapid identification of sterile plates, distinguishing colonies from single or mixed species, and providing immediate alerts for specific pathogens^1^, reducing turnaround time and enhancing patient care.

Our primary objectives are to:

1. train and evaluate multiple object detection architectures (including both two-stage and single-stage detectors) for microbial colony detection on a publicly available dataset, comparing performance and inference speed;
2. curate a new dataset with annotated images of agar plates across a diverse set of culture media and release it publicly;
3. apply transfer learning and Weighted Box Fusion ensemble methods, and evaluate cross-subset generalization as an alternative to subset-specific fine-tuning;
4. quantify statistical uncertainty through bootstrap confidence intervals and assess potential sources of bias in dataset construction and model evaluation.

This paper is structured as follows: the Literature Review surveys existing research and identifies gaps; the Methods section details the datasets, experimental setup, model architectures, and evaluation framework; Results presents the findings with statistical uncertainty estimates; and the Discussion contextualizes these results, examines bias and limitations, and compares with existing work.

## Literature review

Deep learning has been increasingly explored in clinical microbiology, yet colony-level analysis from agar plate photographs remains a relatively narrow subfield. Existing work addresses colony localisation and multi-class detection^11,12^, colony counting via detection or density-map regression^13–15^, and synthetic image generation to mitigate annotation scarcity^16–19^. Direct comparison across studies is complicated by differences in dataset splits, inference pipelines, and evaluation thresholds.

The AGAR dataset, introduced by Majchrowska et al., is a large public dataset for multi-class microbial colony detection and has become a recurrent benchmark in this field^11^. Early comparative studies on AGAR evaluated detectors such as Faster R-CNN, Cascade R-CNN, and YOLOv4, showing that Cascade R-CNN with an HRNet backbone achieved the strongest performance among the original two-stage baselines, while YOLOv4 also performed competitively under the reported evaluation setting^11,12^. Subsequent work reported higher results under modified protocols. These included the combination of style-transfer augmentation with transformer-based feature extraction, as well as the evaluation of more recent YOLO variants under the same augmented setting^20^. More recent work also showed that SAM-based copy-paste augmentation can approach the performance obtained with real training data while using only a fraction of the labelled set^18^. Taken together, these studies suggest that data strategy can be as important as detector architecture.

Beyond AGAR, the dataset landscape has broadened. One public dataset introduced a more heterogeneous evaluation setting, comprising 369 images with 56,865 annotated colonies across 24 species, collected under routine laboratory conditions with variation in smartphone cameras and backgrounds^21^. More recent detector designs have explicitly targeted such dense and heterogeneous settings, including micro-colony detection, while also highlighting persistent difficulties with background variation and very small or adherent colonies^22^. In parallel, recent few-shot work has explored diffusion-based inpainting as a strategy to improve robustness to image corruptions on AGAR^19^.

Overall, prior work has largely relied on a single dataset or on restricted subsets of available benchmarks, with primary emphasis on architecture comparison rather than systematic evaluation of robustness across imaging conditions or the combined effect of training strategies. These limitations motivate the present study, which compares two-stage and single-stage detectors under fixed evaluation settings, quantifies the effect of background variation through transfer learning and cross-subset evaluation, applies WBF ensembling, and tests generalisation on a small curated dataset spanning four culture media not represented in AGAR.

## Methods and experiments

This section describes the two datasets used in this study, the data processing workflow, the model architectures, evaluation metrics, and the reproducibility measures adopted.

### Datasets

#### AGAR dataset

The first part of this study was developed upon the Annotated Germs for Automated Recognition (AGAR) dataset that was provided by Majchrowska, Pawłowski, et al.^11^. The authors’ main objective for creating this dataset was to have a diverse dataset upon which the broader research community could build and improve the field of neural networks application in microbiology. The AGAR dataset comprises 18 thousand annotated photographs of Petri dishes, with over 330 thousand labelled microbial colonies. It includes colonies belonging to four different bacterial species (*Staphylococcus aureus*, *Bacillus subtilis*, *Pseudomonas aeruginosa*, *Escherichia coli*), alongside one yeast strain (*Candida albicans*), inoculated in Trypticase Soy Agar (TSA), a nutrient-rich, non-selective and non-differential medium^11^.

**Fig. 1:**
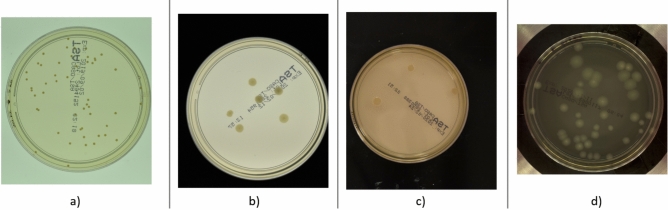
Background settings in the AGAR dataset: (**a**) Bright, (**b**) Dark, (**c**) Vague, (**d**) low resolution^11^.

Photographs are categorized by imaging conditions: high-resolution images captured with white plexiglass (bright set), black plexiglass (dark set), or ambient lighting (vague set), and a low-resolution group^11^. Figure 1 illustrates the various background settings.

The dataset comprises images featuring countable colonies, images depicting empty plates, and images showcasing uncountable colonies, where the enumeration of individual colonies is unfeasible.

We selected images containing colonies of *S. aureus*, *P. aeruginosa*, and *E. coli*, motivated by the greater class balance among these species and their higher clinical significance as prevalent human pathogens. Images featuring *B. subtilis*, *C. albicans*, “Contamination”, or “Defect” annotations were excluded. Furthermore, images with uncountable colonies or more than 100 annotations were also removed. The resulting dataset comprises a total of 9851 images, 182864 annotations across three classes. Of these, 1217 images feature empty plates. By background type, it contains 4466 images from Dark set (45.3%), 3709 Low Resolution (37.7%), 868 from Vague set (8.8%) and 808 from Bright set (8.2%).

**Fig. 2:**
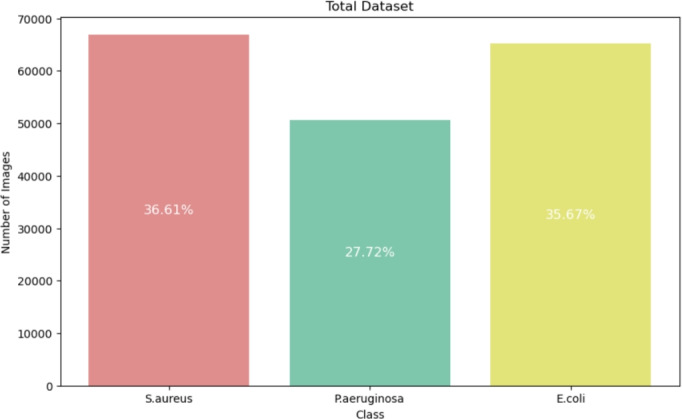
Distribution of classes across the entire dataset. The distribution is near-uniform, with *P. aeruginosa* making up the fewest annotations.

**Fig. 3:**
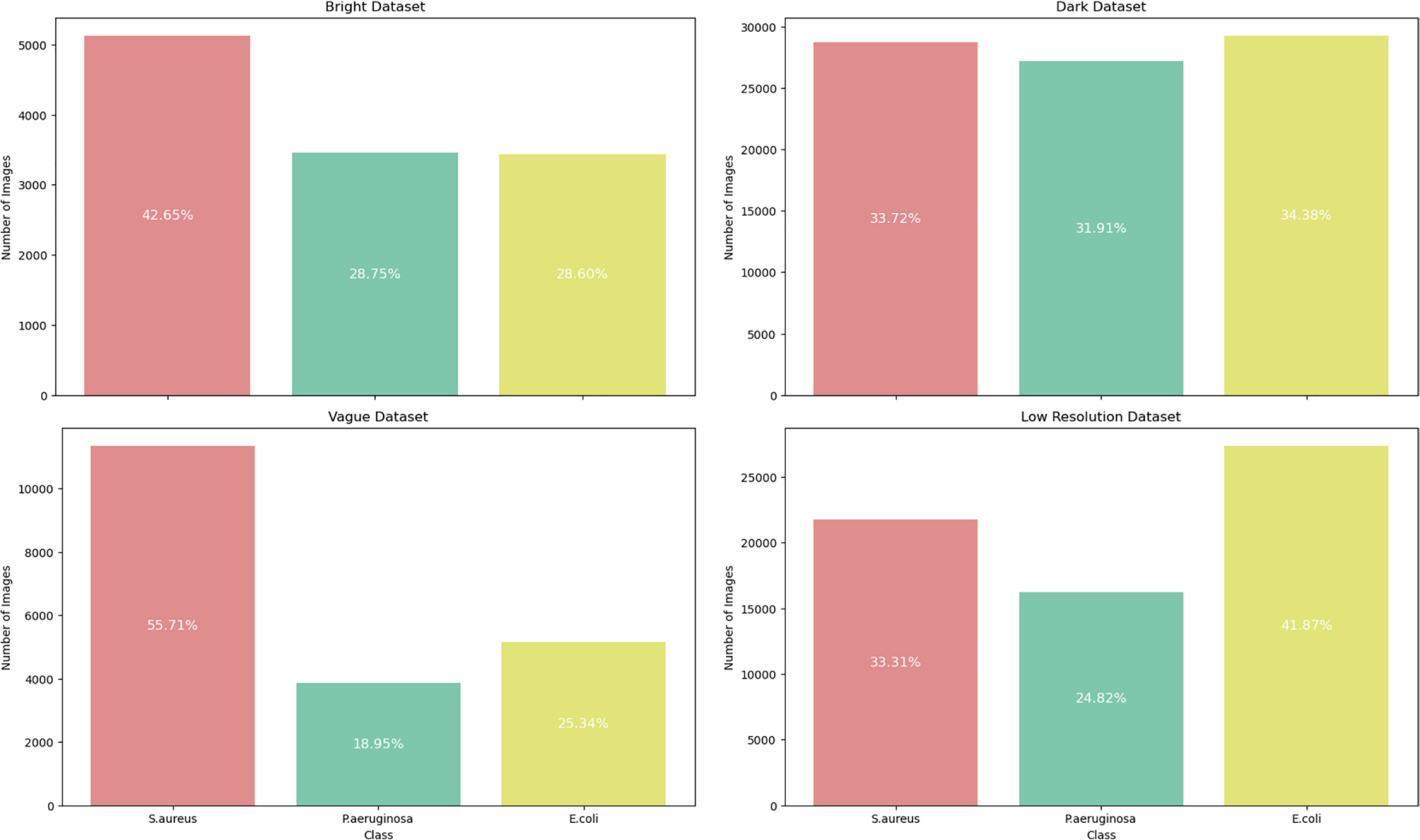
Distribution of classes within each background subset. The dark subset has the most balanced distribution, while the vague subset is the most imbalanced, with over 50% *S. aureus* annotations.

Figures 2 and 3 show the class distribution across the entire dataset and within each background subset. The dataset shows near-uniform class distribution overall, though some imbalance exists within individual subsets (the vague subset, for instance, has over 50% *S. aureus* annotations).

#### Curated dataset

**Fig. 4:**
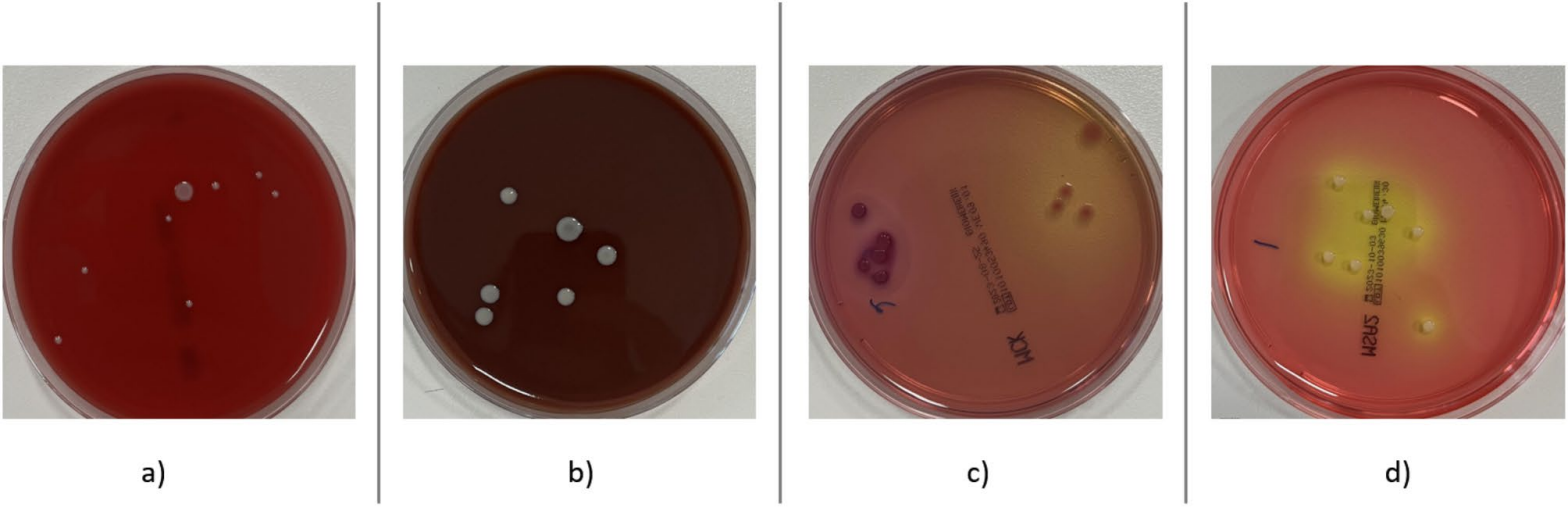
Different culture media in the curated dataset: (**a**) blood agar, (**b**) chocolate agar, (**c**) MacConkey agar, (**d**) Mannitol salt agar.

For the second part of the study, we created a dataset of annotated agar plate images with colonies of *S. aureus*, *P. aeruginosa*, and *E. coli*. We used four types of agar media: blood agar (Fig.  4a) and chocolate agar (Figure 4b), both enriched and non-selective; MacConkey agar (Fig.  4c), a selective and differentiating medium supporting only gram-negative species; and Mannitol salt agar (Fig. 4d), selective and differential for *S. aureus*.

Colonies previously identified by Matrix-Assisted Laser Desorption/Ionization Time-of-Flight (MALDI-TOF) were inoculated onto plates after serial dilutions to produce well-separated colonies. The different culture media exhibit distinct colours, and bacteria react differently depending on the medium they were inoculated on. Even within the same species, slight variations in morphology may arise due to the type of medium utilized. This diversity makes the dataset more reflective of real laboratory conditions than single-medium datasets.

Images were captured using an iPhone 13 mini under consistent (but not standardized) lighting conditions, without specialized equipment for light control. Given the variation in transparency across media, photographs were primarily taken against a dark background. Images were cropped to show only the plates, then uploaded and annotated using Roboflow. Annotations were performed by the first author, following the identification by MALDI-TOF. No formal inter-annotator agreement was computed. The resulting dataset comprises 165 images and 1801 annotations, with 4 images of empty plates. It is publicly available at Zenodo^23^.

**Fig. 5:**
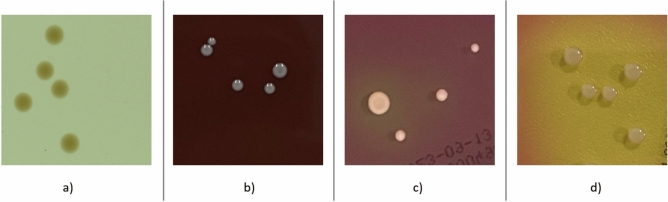
*S. aureus* colonies: (**a**) AGAR dataset, (**b**) blood agar, (**c**) Mannitol salt agar, (**d**) Mannitol salt agar. Colonies are typically circular, smooth, raised, and glistening. On Mannitol salt agar they exhibit golden-yellow pigmentation; on TSA and blood agar they appear opaque, greyish-to-yellow.

**Fig. 6:**
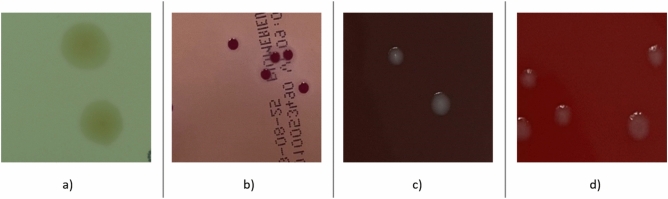
*E. coli* colonies: (**a**) AGAR dataset, (**b**) MacConkey agar, (**c**) chocolate agar, (**d**) blood agar. Colonies are large, circular, and grey on TSA, blood, and chocolate agar. On MacConkey agar they acquire a pink colour due to lactose fermentation.

**Fig. 7:**
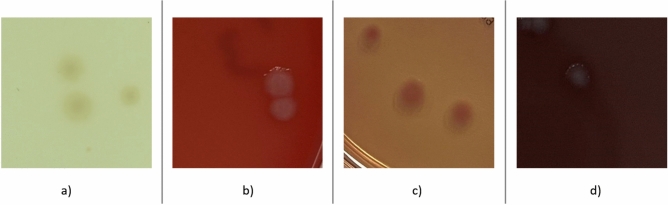
*P. aeruginosa* colonies: (**a**) AGAR dataset, (**b**) blood agar, (**c**) MacConkey agar, (**d**) chocolate agar. Colonies are larger, circular, flat, with irregular margins. On blood and chocolate agar they show greyish pigmentation; on TSA they appear paler. On MacConkey agar they are colourless (non-lactose-fermenting).

Figures 5, 6 and 7 illustrate morphological differences across species and media. Sub-figure (a) in each shows examples from the AGAR dataset for comparison.

### Training workflow and model architectures

**Fig. 8:**
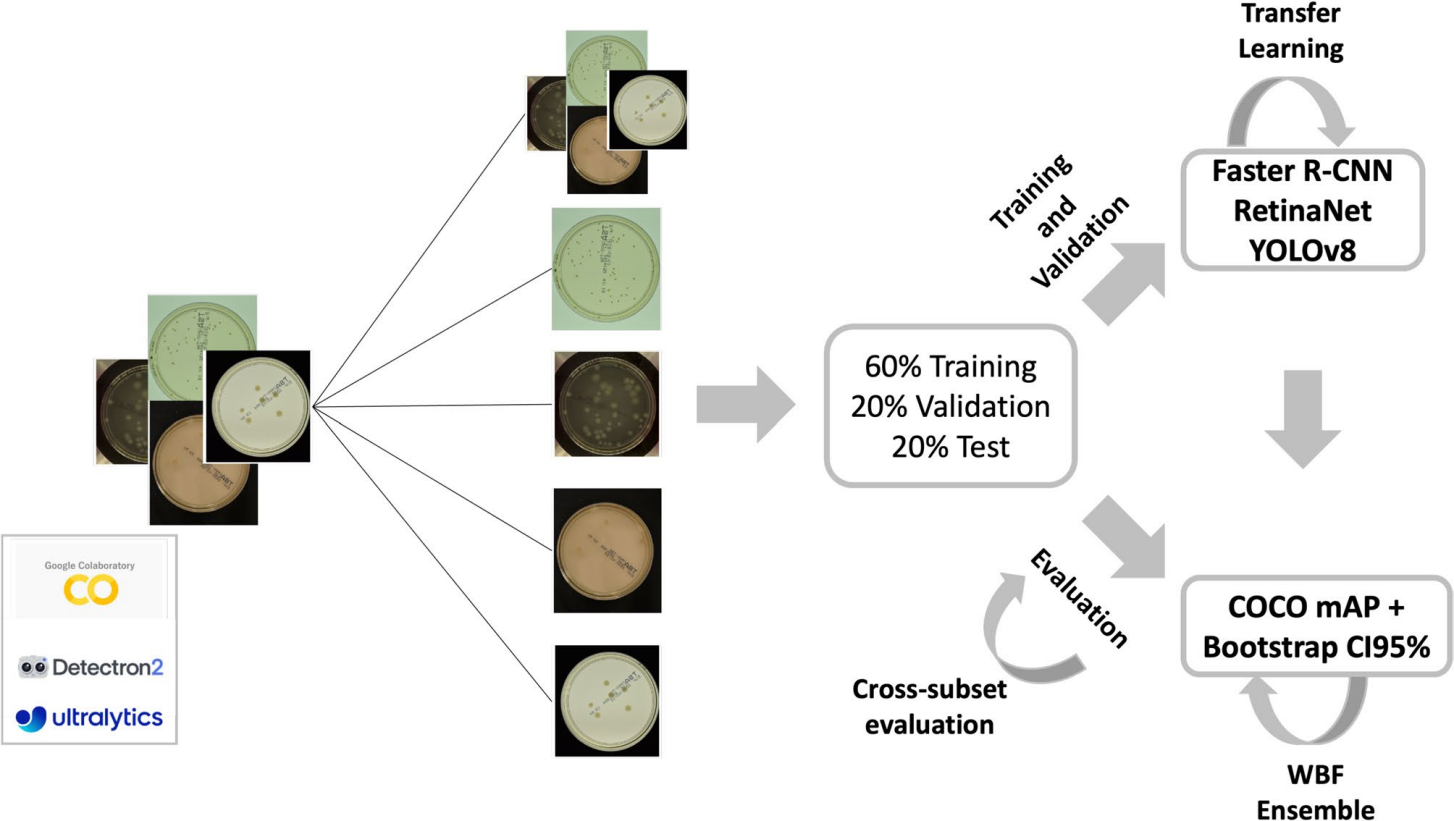
Training workflow.

A schematic view of the workflow is presented in Fig. 8.

#### Detectron2 models

The Detectron2 framework^24^, an open-source library from Facebook AI Research implemented in PyTorch, was used to train two object detection architectures: the two-stage Faster R-CNN and the single-stage RetinaNet, each with ResNet-50 and ResNet-101 backbones and Feature Pyramid Networks (FPN). Models were initialized from Detectron2 model-zoo detection checkpoints. Training was conducted on Google Colab Pro with a T4 GPU.

Each dataset was split into training (60%), validation (20%), and test (20%) subsets. For Detectron2 training, the AGAR dataset was trained for 10 epochs with batch size 8, while the curated dataset was trained for 100 epochs due to its smaller size. Training used stochastic gradient descent (SGD) with momentum 0.9 and weight decay 0.0005. The base learning rate was set to 0.005, with linear warm-up at the start of training, followed by a single step decay during training. Validation metrics were recorded during training, and the test set was reserved for final evaluation. The AGAR dataset was also trained by subset (bright, dark, vague, lowres) in addition to the full total set. Training of the curated dataset was conducted on the full set only.

Full hyperparameter settings and Detectron2 configuration files are provided in the code repository^25^.

#### YOLOv8

To provide a single-stage comparison, we trained YOLOv8 models using the Ultralytics framework. Three model sizes were evaluated on the AGAR dataset: YOLOv8s (small), YOLOv8m (medium), and YOLOv8l (large), with YOLOv8m selected as the primary model. Unlike the Detectron2 experiments, YOLOv8 was trained only on the complete AGAR dataset rather than on the individual subsets. AGAR models were trained for up to 100 epochs with early stopping (patience 20), a batch size of 8, and the Ultralytics default optimization pipeline. For the curated dataset, the same training protocol was followed with up to 1000 epochs (patience 200), including an additional transfer-learning experiment initialized from AGAR-trained weights. All experiments used a fixed random seed (seed = 42) for reproducibility. YOLOv8 was trained on the total AGAR dataset only, not on individual subsets, due to computational constraints. The Detectron2 cross-subset experiments showed that subset-specific training offers minimal gains over a single total-trained model, supporting this choice.

Full YOLOv8 training configurations are documented in the code repository^25^.

#### Transfer learning and cross-subset evaluation

Two strategies for improving per-subset and cross-dataset performance were compared.

**Transfer learning** was applied to evaluate whether models trained on the full AGAR dataset could improve performance on individual subsets and on the curated dataset. For the AGAR dataset, per-subset models that performed below the dataset-wide model were retrained using the best-performing model’s weights as initialization. For the curated dataset, the best-performing AGAR model was used to initialize a second training phase on the curated images. In both cases, all other hyperparameters remained unchanged. Retrained models were evaluated under the same protocol to quantify the effect of transfer learning relative to training from Detectron2 model-zoo checkpoints alone.

**Cross-subset evaluation** tested a simpler alternative: the model trained on the full AGAR dataset was evaluated directly on each subset’s test set without any fine-tuning. Comparing the two approaches isolates the contribution of fine-tuning from the generalization already provided by training on diverse data. Separately, the best-performing AGAR model was also evaluated directly on the curated test set without fine-tuning, to quantify the cross-domain gap between the two datasets.

#### Ensemble: weighted box fusion

Weighted Box Fusion (WBF)^26^ was used to aggregate predictions from multiple trained models. WBF leverages confidence scores from proposed bounding boxes to generate averaged fused boxes. Three configurations were evaluated: (1) mixed ensembles combining YOLOv8 and Detectron2 predictions; (2) Detectron2-only ensembles; and (3) YOLOv8-only ensembles. For each subset, a grid search over IoU thresholds, skip-box thresholds, and weighting schemes (uniform and AP-based) was performed, retaining the best-performing configuration.

### Evaluation metrics

We adopted the Common Objects in Context (COCO) evaluation metrics^27^, specifically the mAP across various Intersection over Union (IoU) thresholds, and the mean Average Recall (mAR).

To evaluate both the position and the class of a predicted bounding box, IoU is computed between the ground truth box and the predicted box. IoU measures the overlap as a proportion of the intersected area to the combined area of both boxes, and it is calculated using the Jaccard similarity coefficient 1. A higher IoU indicates better localization, with a perfect prediction resulting in an IoU of 1 (or 100%). The Jaccard similarity coefficient (IoU) measures localization quality:

1 $$\begin{aligned} \text {IoU} = \frac{|A \cap B|}{|A \cup B|} \end{aligned}$$

Precision and recall are defined as:

2 $$\begin{aligned} \text {Precision} = \frac{\text {TP}}{\text {TP} + \text {FP}}, \quad \text {Recall} = \frac{\text {TP}}{\text {TP} + \text {FN}} \end{aligned}$$

Average Precision (AP) is computed from the precision-recall curve at 101 recall thresholds:

3 $$\begin{aligned} \text {AP} = \frac{1}{101} \sum _{r \in \{0.00, 0.01, \ldots , 1.00\}} p(r) \end{aligned}$$

The mAP is the mean of AP across all *k* classes:

4 $$\begin{aligned} \text {mAP} = \frac{1}{k} \sum _{i=1}^{k} \text {AP}_i \end{aligned}$$

The COCO dataset calculates mAP at various IoU thresholds, typically ranging from 0.5 to 0.95 in increments of 0.05. The final mAP is the average of the mAP values across these different thresholds. This approach is favored for its ability to provide a detailed, fine-grained evaluation of models across varying levels of localization accuracy^27^.

All evaluations used a confidence threshold of 0.0 before COCO evaluation. This follows the standard COCO protocol, in which detections are ranked by confidence and average precision is computed from the full precision-recall curve over fixed recall thresholds, rather than at a single confidence operating point^27,28^. Consequently, mAP reflects ranking quality across predictions, not an arbitrary score cutoff^28^. Ultralytics YOLO uses a similarly minimal validation threshold (conf = 0.001) for precision-recall curve computation, serving the same practical purpose^29^. These metrics collectively provide a robust framework for assessing model performance, balancing precision, recall, and localization accuracy^30^.

To provide confidence intervals for all primary metrics, we performed stratified bootstrap resampling of the test set^31^. For each configuration, COCO evaluation was recomputed on bootstrap samples drawn with replacement: 300 samples for the best-performing AGAR models, 100 for the remaining Detectron2 configurations, and 1000 for the curated dataset (reflecting its smaller size and higher statistical uncertainty). We report 95% percentile confidence intervals (CI) (2.5th and 97.5th percentiles).

## Results

Results are reported in terms of mean Average Precision (mAP), mean Average Recall (mAR), and per-class AP for *S. aureus*, *P. aeruginosa*, and *E. coli*. The experiments address five questions: (1) how do Detectron2 and YOLOv8 architectures compare on this task; (2) how do imaging conditions (background type) affect detection performance; (3) how does performance vary across bacterial species and datasets; (4) does fine-tuning on individual subsets improve over a single model trained on diverse data; and (5) do cross-architecture ensembles outperform single-model predictions.

### AGAR dataset

#### Model evaluation

Table 1 summarizes detection results on the AGAR dataset. Detectron2 models were trained separately for each subset; YOLOv8m was trained on the total dataset only (cross-subset results are reported in Table 2).

On the total dataset, YOLOv8m achieved the highest mAP at 69.0% (95% CI: 67.0-70.3), outperforming the best Detectron2 model (Faster R-CNN R-101, 63.1%, 95% CI: 62.1-63.9) by 5.9 percentage points (pp) with non-overlapping confidence intervals. The gap was particularly pronounced for *S. aureus*: YOLOv8m reached 60.5% (95% CI: 56.5-64.2) compared to 53.6% (95% CI: 51.7-55.1) for Faster R-CNN R-101, a 6.9 pp difference.

Among Detectron2 models, Faster R-CNN consistently outperformed RetinaNet across all subsets, particularly for *S. aureus*. On the total dataset, Faster R-CNN R-101 achieved 53.6% for this class while RetinaNet R-101 reached 38.4%.

Across subsets, Bright subset was the most challenging for Detectron2 (best mAP: 54.1%, Faster R-CNN R-101), while Dark and Low Resolution performed closer to the total dataset. The Vague subset showed intermediate difficulty, with RetinaNet *S. aureus* performance dropping below 25%.

As in per-class evaluation, *E. coli* was generally the strongest class, especially for Faster R-CNN and YOLOv8m, although RetinaNet performance on the Bright subset was notably lower. *P. aeruginosa* showed stable mid-range performance except in the Bright subset, where all models dropped below 49%. *S. aureus* was the most variable and weakest class overall: RetinaNet achieved below 25% on Vague and below 21% on Bright, while Faster R-CNN remained above 40% across all subsets.

Bootstrap confidence intervals are reported for the total dataset, where all five models were evaluated on the same test set; per-subset intervals are available in the reproducibility bundle.

**Table 1:** Detection results (%) on the AGAR dataset by model and subset.

Subset	Model	Backbone	mAP	*S. aureus*	*P. aeruginosa*	*E. coli*
Total	Faster R-CNN	R-50	62.63 [61.9-63.4]	53.87 [52.2-55.3]	64.85 [63.3-66.1]	69.18 [68.4-70.2]
	Faster R-CNN	R-101	63.07 [62.1-63.9]	53.60 [51.7-55.1]	65.34 [63.9-66.6]	70.27 [69.3-71.3]
	RetinaNet	R-50	56.39 [55.3-57.4]	37.26 [35.3-39.3]	62.88 [61.1-64.7]	69.03 [67.8-70.2]
	RetinaNet	R-101	56.44 [55.4-57.3]	38.41 [36.3-40.3]	62.27 [60.8-64.0]	68.64 [67.6-69.8]
	YOLOv8m	-	**69.01** [67.0-70.3]	**60.47** [56.5-64.2]	**71.09** [68.1-72.7]	**75.48** [73.4-77.5]
Bright	Faster R-CNN	R-50	51.39	50.80	44.56	58.82
	Faster R-CNN	R-101	**54.07**	**51.30**	**48.77**	**62.15**
	RetinaNet	R-50	41.72	20.93	44.71	59.54
	RetinaNet	R-101	40.06	19.86	40.85	59.48
Dark	Faster R-CNN	R-50	63.04	**55.65**	64.42	69.04
	Faster R-CNN	R-101	**63.29**	54.43	**65.81**	**69.61**
	RetinaNet	R-50	58.92	43.95	63.79	69.03
	RetinaNet	R-101	58.29	42.97	63.47	68.42
Vague	Faster R-CNN	R-50	53.60	**40.77**	52.81	67.22
	Faster R-CNN	R-101	**54.11**	40.48	53.74	68.11
	RetinaNet	R-50	49.92	27.23	54.54	67.98
	RetinaNet	R-101	49.68	24.72	**55.04**	**69.28**
Low res.	Faster R-CNN	R-50	**63.20**	**52.92**	65.85	70.82
	Faster R-CNN	R-101	62.92	51.67	**66.04**	71.05
	RetinaNet	R-50	57.00	35.62	64.44	70.96
	RetinaNet	R-101	57.83	37.88	64.13	**71.49**

Best values per subset are in bold.

#### Transfer learning vs. cross-subset generalization

Table 2 compares three evaluation strategies for Faster R-CNN R-101 on the AGAR subsets: (1) the baseline model trained on each subset individually, (2) transfer learning from the total-trained model, and (3) direct cross-subset evaluation of the total-trained model without fine-tuning. Transfer learning was applied only to subsets that performed below the total-dataset model; Dark was excluded as it already had the strongest baseline among subsets. YOLOv8m cross-subset results are included for reference.

Transfer learning produced measurable gains over the subset-specific baselines, with the largest improvement on the Bright subset (+5.41 pp). However, cross-subset evaluation of the total-trained model achieved comparable results: the differences between transfer learning and cross-subset ($\Delta$(TL-CS)) ranged from -0.32 pp (Low Resolution, where cross-subset was slightly better) to +1.05 pp (Bright). YOLOv8m, trained only on the total dataset, outperformed both Detectron2 strategies on all subsets. Per-class results and bootstrap confidence intervals for all subsets are available in the reproducibility bundle^32^.

**Table 2:** Comparison of subset optimization strategies for Faster R-CNN R-101 (% mAP).

Subset	Baseline	Transfer learning	Cross-subset	$\Delta$(TL-CS)	YOLOv8m
Bright	54.07	59.48 (+ 5.41)	58.43 (+ 4.36)	+ 1.05	**69.95**
Vague	54.11	57.08 (+ 2.97)	56.98 (+ 2.87)	+ 0.10	**63.70**
Low resolution	62.92	64.62 (+ 1.70)	64.94 (+ 2.02)	– 0.32	**71.88**
Dark	63.29	–	64.22 (+ 0.93)	–	**70.54**

Parenthesis values indicate the gain over baseline. $\Delta$(TL-CS) refers to the difference between transfer learning and cross-subset mAP values. Best values per row in bold.

#### Ensemble results

Table 3 presents the WBF ensemble results. The best mixed ensemble on the total dataset combined four models (three YOLOv8 sizes and Faster R-CNN R-101) with AP-based weights and IoU threshold 0.75. On the AGAR subsets, ensembles selected by grid search consistently combined two models (YOLOv8m and Faster R-CNN R-101). Full ensemble configurations and WBF parameters are reported in the reproducibility bundle^32^.

The mixed ensemble achieved 70.54% mAP on the total dataset (95% CI: 68.4-71.7), a 1.52 pp gain over the best single model (YOLOv8m, 69.01%). The YOLOv8-only ensemble of three model sizes reached 70.04%, while the Detectron2-only ensemble (Faster R-CNN R-50 and R-101) achieved 64.34%. On the total dataset, the mixed ensemble gained 0.50 pp over the YOLO-only ensemble (70.54% vs. 70.04%), with a slightly narrower bootstrap interval (68.4–71.7 vs. 68.1–71.5). On two of four subsets (Bright and Low Resolution), the best single model outperformed the mixed ensemble.

**Table 3:** WBF ensemble results (% mAP) on the AGAR dataset.

Subset	Mixed	YOLO-only	D2-only	Best single
Total	**70.54** [68.4–71.7]	70.04 [68.1–71.5]	63.42 [61.6–65.2]	69.01
Bright	69.07	–	60.32	**69.95**
Dark	**70.63**	–	65.24	70.54
Vague	**64.06**	–	57.84	63.70
Low res.	70.69	–	65.58	**71.88**

95% bootstrap CIs shown in brackets for the total dataset. Mixed refers to YOLOv8 + Detectron2; YOLO-only refers to YOLOv8 s/m/l ensemble (total set only, as no subset-specific YOLO models were trained); D2-only refers to Detectron2 models only. Best single is YOLOv8m in all cases (total-trained, evaluated per subset via cross-subset evaluation). Best values per row in bold.

### Curated dataset

Table 4 summarizes the curated-dataset results. Among the Detectron2 models, RetinaNet R-50 achieved the highest mAP (52.41%), followed by RetinaNet R-101 (51.96%), Faster R-CNN R-50 (51.27%), and Faster R-CNN R-101 (49.84%).

YOLOv8m trained directly on the curated dataset achieved 54.93% mAP. Initializing YOLOv8m from AGAR-trained weights increased performance to 55.41%. The AGAR-trained YOLOv8m model evaluated directly on the curated test set without fine-tuning achieved 9.03% mAP.

The mixed WBF ensemble achieved 58.65% mAP (95% CI: 57.06-63.73). This corresponds to a gain of 3.24 pp over the best transferred single model (YOLOv8m initialized from AGAR, 55.41%), 3.72 pp over the best direct single model (YOLOv8m trained on curated only, 54.93%), and 6.24 pp over the best Detectron2 baseline (RetinaNet R-50, 52.41%) (Table 5).

**Table 4:** Baseline detection results (%) on the curated dataset.

Model	Backbone	mAP	*S. aureus*	*P. aeruginosa*	*E. coli*
Faster R-CNN	R-50	51.27 [49.15-55.93]	53.40 [50.48-62.03]	44.47 [37.42-50.52]	55.94 [51.80-63.15]
Faster R-CNN	R-101	49.84 [47.83-55.31]	49.92 [46.65-59.07]	44.69 [39.93-51.91]	54.91 [51.39-63.41]
RetinaNet	R-50	52.41 [50.56-57.51]	52.22 [49.77-61.29]	47.78 [42.85-53.37]	57.23 [52.64-66.47]
RetinaNet	R-101	51.96 [49.81-57.52]	51.44 [47.75-62.93]	46.70 [42.50-51.25]	57.73 [52.87-66.16]
YOLOv8m	–	**54.93** [53.17-60.57]	**54.37** [52.42-63.11]	**50.37** [46.34-55.70]	**60.05** [55.04-70.61]

Point estimates are taken from the stored evaluation outputs, and 95% bootstrap confidence intervals are shown in brackets. Best point estimate per column is shown in bold.

**Table 5:** Transfer learning and ensemble results (%) on the curated dataset.

Configuration	mAP	$\Delta$ vs. baseline
Transfer learning (from AGAR weights)		
RetinaNet R-50 + TL (dark)	53.59 [51.3-59.3]	+ 1.18
YOLOv8m + TL	55.41 [53.3-60.9]	+ 0.48
AGAR model (no fine-tuning)	9.03 [5.1-17.4]	–
WBF Ensembles		
Mixed (10 models)	**58.65** [57.1-63.7]	+ 3.72
YOLO-only (4 models)	57.69 [56.0-63.1]	+ 2.76
D2-only (5 models)	57.01 [55.2-61.6]	+ 4.60

$\Delta$ vs. baseline denotes the gain over the best direct baseline within each family for the single-family configurations (YOLOv8m: 54.93%; RetinaNet R-50: 52.41%). For the mixed ensemble, $\Delta$ is reported relative to the best direct single model overall (YOLOv8m: 54.93%). Values in brackets denote 95% bootstrap confidence intervals. The AGAR model row reports YOLOv8m evaluated directly on the curated test set without fine-tuning. Best mAP is shown in bold.

## Discussion

### Architecture choice as the primary performance driver

This study compared five object detection configurations across two datasets. The clearest result was that architecture choice was the strongest performance driver in these experiments. On the AGAR total dataset, YOLOv8m outperformed the best Detectron2 model by 5.9 pp (69.0% vs. 63.1%), with non-overlapping bootstrap confidence intervals. YOLOv8m also achieved the highest performance across the AGAR subset evaluations. At the per-class level, the advantage was particularly pronounced for *S. aureus* (60.5% vs. 53.6%), the smallest colony class.

This performance gap is consistent with architectural differences. Detectron2 models use a Feature Pyramid Network (FPN) with anchors at predefined scales (32, 64, 128, 256, 512 px). Bounding-box analysis shows that 82% of *S. aureus* annotations fall below the 64 px anchor level, placing them at the resolution limit of the smallest FPN features. Faster R-CNN partially compensates through its two-stage refinement, but RetinaNet’s single-stage design produces a much larger performance drop on small colonies (*S. aureus*: 38.4% vs. 53.6% for Faster R-CNN on total). YOLOv8’s anchor-free detection head with adaptive multi-scale features handles the scale mismatch more effectively.

Within the YOLOv8 family, the three model sizes (s, m, l) achieved near-identical mAP on AGAR total (68.8%, 69.0%, 68.9%), while inference time varied from 22 ms (s) to 64 ms (l) per image. This plateau indicates that performance is limited by data characteristics rather than model capacity, and that YOLOv8m provides the optimal accuracy-speed trade-off.

Inference speed also differentiates the architectures for practical deployment. YOLOv8 models were approximately 5 times faster than Detectron2: YOLOv8m averaged 40 ms per image on AGAR total compared to 219 ms for Detectron2 models. On the curated dataset (smaller images), these values were 23 ms and 128 ms, respectively. Although the absolute latency differences are unlikely to dominate the end-to-end laboratory workflow, YOLOv8 provided both higher detection accuracy and lower inference latency, making it the most favorable overall configuration in this comparison.

### Training data diversity over subset specialization

The comparison between transfer learning and cross-subset evaluation (Table 2) showed that a single model trained on the full, diverse dataset generalized comparably to individual imaging conditions relative to a model explicitly fine-tuned for each condition.

For Faster R-CNN R-101, the differences between transfer learning and cross-subset evaluation were small: + 1.05 pp on Bright, + 0.10 pp on Vague, and – 0.32 pp on Low-Resolution, where cross-subset evaluation was slightly better. Both strategies improved substantially over the subset-only baselines, indicating that exposure to a more diverse training set was the main contributor to performance, while fine-tuning provided only modest additional gains.

From a deployment perspective, this favors the use of a single model trained on diverse data over maintaining multiple condition-specific models.

### Ensemble methods: complementary gains

WBF ensembles improved over the corresponding single-model baselines on both the AGAR total and curated datasets. On the AGAR total dataset, the mixed ensemble improved mAP by 1.5 pp (70.5% vs. 69.0%). On the curated dataset, the mixed ensemble reached 58.65%, corresponding to a gain of 3.24 pp over the best single model overall (YOLOv8m + TL, 55.41%) and 3.72 pp over the best direct single model (YOLOv8m trained on curated only, 54.93%).

Across both datasets, the mixed ensemble outperformed the single-family ensembles, indicating that combining YOLOv8 and Detectron2 predictions provided more complementary information than combining models from either family alone. Although the Detectron2 models were individually weaker, their inclusion in the mixed ensemble still produced the best overall results.

### Curated dataset and domain shift

The curated dataset presented a more challenging detection setting, combining a much smaller sample size, multiple culture media, and smaller colony bounding boxes (median 59x63 px vs. 112x114 px in AGAR). The AGAR-trained YOLOv8m model, applied directly to the curated test set, achieved only 9.0% mAP, indicating that direct transfer from AGAR to the curated dataset was insufficient without additional domain-specific adaptation.

Fine-tuning on the curated dataset increased performance to 55.4% for YOLOv8m with AGAR initialization, and the mixed ensemble reached 58.7%.

The performance gap between architectures was narrower on the curated dataset (YOLOv8m: 55.4% vs. RetinaNet R-50: 52.4%) than on AGAR (69.0% vs. 63.1%). This narrower margin may reflect the limited size of the curated dataset, together with the differences in visual characteristics between the two datasets, including colony appearance and scale. In this sense, the curated dataset should be interpreted primarily as a comparison setting for probing domain shift, rather than as a large standalone benchmark designed to support definitive ranking of model families.

### Comparison with prior work

On the AGAR benchmark introduced by Majchrowska et al., the main five-class baseline study reports mAP values from 49.3% to 59.4%, with Cascade R-CNN+HRNet achieving 52.0% on the higher-resolution subset and 59.4% on the lower-resolution subset^11^. In that protocol, training and inference are patch-based, using 512x512 crops with overlap and post-processing to merge predictions at whole-image level. By contrast, our study uses a full-frame pipeline on an AGAR-derived three-class subset excluding images with more than 100 annotations. Because of these substantial differences, direct numerical comparison should be interpreted with caution.

Within our protocol, the best single model (YOLOv8m) reaches 69.0% mAP and a mixed WBF ensemble achieves 70.5%. These values are numerically higher than the published five-class AGAR baselines, but they are obtained under a reduced three-class setting with additional filtering and are therefore not directly comparable to the original AGAR benchmark.

Table 6 summarises recent colony-detection results on AGAR and related datasets. Among AGAR-based studies, recent reports indicate that stronger feature-pyramid and YOLO-based variants can approach or exceed 70% mAP under modified training and evaluation settings, including higher-resolution validation pipelines and synthetic or style-transfer-based augmentation. Non-AGAR studies are included only as contextual evidence that YOLO-based detectors also transfer well to other colony-detection domains.

**Table 6:** Comparison with prior work.

Study	Dataset	Best model	mAP	Protocol note
AGAR-based studies				
Majchrowska et al.^11^	AGAR HR/LR (5)	Cascade R-CNN +HRNet	52.0 / 59.4	512x512 crops; overlap-merging
Majchrowska et al.^12^	AGAR HR subset (5)	YOLOv4	52.9	Crop-based; countable bright/dark subset
Ebert et al.^33^	AGAR HR val. (5)	TOOD+AttnPAFPN	70.4	1536x1536; validation set
Yang et al.^20^	AGAR (5)	YOLOv8x	76.7	Style-transfer- expanded dataset
Mennemann et al.^18^	AGAR (5)	YOLOv8n	64.9	800x800; val. set; low-data study
Related AGAR augmentation study				
Pawłowski et al.^16^	AGAR HR test set (5)	Cascade R-CNN+HRNet	41.6	Trained on synthetic style-transfer data
Non-AGAR studies, context only				
Wang et al.^22^	ADBC / MBCD	Colony-YOLO	91.1 / 96.1	Different datasets; improved YOLOv8n variant
This study				
This study	AGAR-derived subset (3)	Faster R-CNN R-101	63.1	Full-frame; images with > 100 annotations excluded
This study	AGAR-derived subset (3)	YOLOv8m	69.0	Best single model
This study	AGAR-derived subset (3)	Mixed WBF (4 models)	70.5	Best ensemble

### Bias analysis

**Exclusion of plates with > 100 annotations.** This threshold was applied to reduce noise from heavily confluent plates where individual colony boundaries become ambiguous. To quantify the impact, we conducted a stress test evaluating all four Detectron2 models on the excluded images, stratified by density. Performance declined when increasing annotations number: at 101-150 annotations, Faster R-CNN R-101 achieved 54.7% mAP (vs. 63.1% on the total test set); at 151-300 annotations, it dropped to 44.5%. RetinaNet showed a sharper decline from 46.2% at 101-150 annotations to 33.1% at 151-300. The full stress test results are available in the reproducibility bundle.

**Anchor-size mismatch.** Bounding-box analysis across both datasets revealed a systematic scale mismatch between colony sizes and the default FPN anchor configuration. In the AGAR dataset, *S. aureus* colonies have a median size of 47x47 px, with 14.3% falling below the smallest anchor level (32 px) and 82.0% below 64 px. By contrast, *E. coli* (median 162x166 px) and *P. aeruginosa* (median 141x141 px) are well-covered by mid-range anchors. The curated dataset shows a similar pattern with overall smaller objects (median 59x63 px, 54% below 64 px). This analysis explains both the consistent difficulty of *S. aureus* detection and the lower absolute mAP on the curated dataset. No anchor rescaling or small-object-oriented loss modifications were attempted, which is a limitation of this work; however, YOLOv8’s anchor-free design inherently bypasses this constraint, which partly accounts for its superior *S. aureus* performance. The full bounding-box statistics and anchor coverage tables are provided in the reproducibility bundle.

**Background-based subset division.** Training subsets separately by background type was a deliberate experimental choice to isolate the effect of imaging conditions on detection performance. The cross-subset evaluation (Table 2) shows that a single model trained on the combined dataset generalizes well across conditions, suggesting the subset division is informative for analysis but not required for deployment.

**Class distribution.** While the overall AGAR dataset is near-uniformly distributed across the three retained species, the vague subset has substantial *S. aureus* overrepresentation. No class-rebalancing strategies were applied in this subset, which is a limitation.

**Curated dataset size and annotation.** The curated dataset is small, comprising 165 images and 1,801 annotations. Bootstrap confidence intervals on this dataset are approximately twice as wide as those on AGAR, indicating greater statistical uncertainty. Annotations were produced by a single expert, and there was no inter-annotator validation. However, the annotator is a clinical pathologist with routine experience in colony identification, and labels were assigned based on prior MALDI-TOF identification of the corresponding colonies. Future extensions should include a larger dataset and multi-annotator validation.

**Fig. 9:**
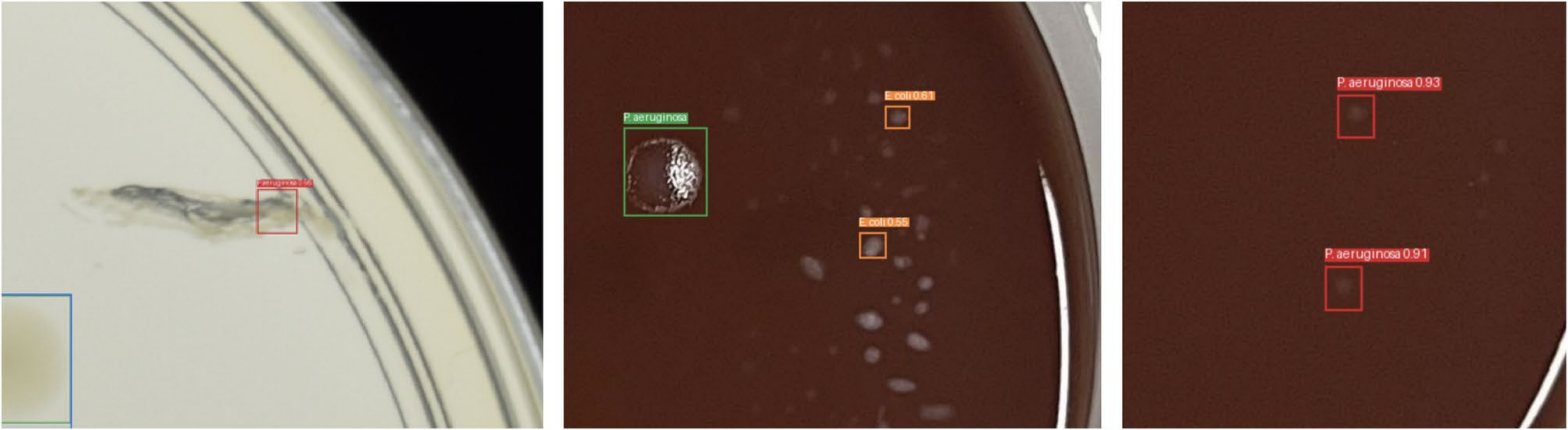
Examples of false-positive detections on AGAR and curated dataset images.

**Imaging conditions for the curated dataset.** The curated dataset was acquired without a standardized imaging setup, with no light box, no fixed camera-to-plate distance, and no controlled ISO or exposure settings. As a result, reflections and variation in background contrast introduced additional image variability. In addition, the inoculation procedure, based on serial dilution to obtain well-separated colonies, differs from routine clinical practice, where plates typically exhibit denser and more heterogeneous growth. Figure 9 shows representative false-positive detections, highlighting how reflections and background patterns can contribute to visually ambiguous regions. Future work should adopt a standardized acquisition protocol with fixed ISO, exposure, and camera-to-plate distance.

### Limitations

Beyond the biases discussed above, several limitations apply. First, only three of the five species in the AGAR dataset were used; results do not generalize to *B. subtilis* or *C. albicans*. Second, the curated dataset uses a non-clinical inoculation protocol, limiting direct clinical applicability. Third, while we report COCO mAP (IoU 0.50-0.95), the clinical relevance of precise bounding-box localization (as opposed to mere detection at IoU 0.50) for culture plate reading has not been established. Fourth, no ablation was performed on anchor configurations or loss functions for small-object detection, which could further improve *S. aureus* performance. Fifth, YOLOv8 was trained only on the full AGAR dataset, not on individual subsets; while the Detectron2 experiments demonstrate that cross-subset and transfer learning yield near-identical results, a YOLO-specific comparison could have confirmed this pattern. Sixth, the study evaluates detection performance using object-detection metrics rather than downstream clinical or counting-oriented endpoints, so the relationship between higher mAP and practical laboratory benefit remains indirect. Seventh, the study used YOLOv8 as the YOLO-family representative; subsequent versions may yield different performance characteristics. Eighth, all inference benchmarks were conducted on a single GPU type (Google Colab T4), and absolute latencies will differ on other hardware.

## Conclusion

This study evaluated five object detection architectures for bacterial colony classification on agar plates across two datasets and multiple imaging conditions. Three main findings emerged.

First, architecture choice was the dominant factor: YOLOv8m outperformed all Detectron2 models on the AGAR dataset, with an additional 5x speed advantage relevant for laboratory throughput.

Second, training data diversity proved more valuable than subset-specific optimization: a single model trained on the full dataset generalized as well as explicitly fine-tuned models, simplifying potential deployment pipelines.

Third, cross-architecture WBF ensembles provided consistent but diminishing gains over the best single model (1.5 pp on AGAR, 3.2 pp on the curated dataset), with the largest benefit coming from architectural diversity rather than model count.

The curated dataset, featuring four culture media types and three bacterial species, is publicly released alongside all code, training configurations, model weights, and a complete results bundle. Bootstrap confidence intervals, anchor analysis, stress testing on dense plates, and inference speed benchmarks provide the methodological transparency required for reproducibility and future benchmarking.

Practical deployment in clinical microbiology will require larger, multi-site annotated datasets with standardized imaging protocols. The models could serve as annotation-assistance tools to accelerate dataset curation, a more realistic near-term application than full automation of culture plate reading.

## Supplementary Information

Supplementary Information.

## Data Availability

The curated dataset is publicly available on Zenodo (DOI: 10.5281/zenodo.18505210). The AGAR dataset is available at https://agar.neurosys.com/11. The complete results bundle (all model predictions, evaluation outputs, bootstrap reports, anchor analysis, stress test results, inference speed benchmarks, and WBF search logs) is archived on Zenodo (DOI: 10.5281/zenodo.19048988). Trained model weights are archived on Zenodo (DOI: 10.5281/zenodo.18922895). All code, training configurations, evaluation scripts, and reproducibility instructions are archived on Zenodo (DOI: 10.5281/zen- odo.19065200) and available on GitHub: https://github.com/jozedu/micro-colony-detection-reproducibility-package

## Code availability

All code, training configurations, evaluation scripts, and reproducibility instructions are archived on Zenodo (DOI: 10.5281/zenodo.19065200) and available on GitHub: https://github.com/jozedu/micro-colony-detection-reproducibility-package.
