# Supplementary material for: Deep neural networks in medical microbiology for bacterial colonies classification
Source: Sci Rep. 2026 May 12;16:21683. doi: 10.1038/s41598-026-48621-0 (PMC13357578; doi:10.1038/s41598-026-48621-0)
Supplement: Supplementary file 1 — Supplementary Information. [file 41598_2026_48621_MOESM1_ESM.pdf]

Supplementary Information

Deep Neural Networks in Medical Microbiology for Bacterial Colonies Classification

José Duarte Pereira, Bruno Veloso, João Gama

This document provides supplementary analyses referenced in the main manuscript. Complete evaluation outputs, predictions, and raw data are available in the paper results bundle archived on Zenodo (DOI: 10.5281/zenodo.19048988). The code repository is archived on Zenodo (DOI: 10.5281/zenodo.19065200), trained model weights on Zenodo (DOI: 10.5281/zenodo.18922895), and the curated dataset on Zenodo (DOI: 10.5281/zenodo.18505210).

1 YOLOv8 Model Size Comparison

Three YOLOv8 model sizes (small, medium, large) were trained on the full AGAR dataset under identical conditions to evaluate whether increased model capacity improves detection performance. Table 1 reports the results.

**Model**	**mAP**	***S. aureus***	***P. aeruginosa***	***E. coli***	**Speed (ms)**
YOLOv8s	68.78	60.06	70.79	75.51	22
YOLOv8m	69.01	60.47	71.09	75.48	40
YOLOv8l	68.89	60.70	70.67	75.31	64

Table 1: YOLOv8 model size comparison on AGAR total test set. Detection metrics (%) and mean inference time (ms/image) on Google Colab T4 GPU.

The mAP difference across the three sizes is 0.23 pp (68.78-69.01%), and per-class results are similarly stable. Inference time scales linearly with model size: YOLOv8l is approximately 3x slower than YOLOv8s. YOLOv8m was selected as the primary model for subsequent experiments based on the accuracy-speed balance.

2 Inference Speed Benchmark

All models were benchmarked on both datasets using a Google Colab T4 GPU. Table 2 reports mean inference time per image.

The speed difference reflects both architectural design (single-stage anchor-free vs. two-stage or anchor-based) and image resolution. Within Detectron2, RetinaNet is slightly faster than Faster R-CNN at the same backbone depth, and R-50 is consistently faster than R-101. Within YOLOv8, the expected size trend holds cleanly.

3 Anchor Coverage Analysis

The Detectron2 models use a Feature Pyramid Network (FPN) with anchors at five predefined scales: 32, 64, 128, 256, and 512 px. Bounding-box analysis was performed to quantify the proportion of annotations falling below each anchor level. Tables 3 and 4 report the cumulative percentage of annotations with bounding-box area below each anchor area threshold (anchor^2), alongside median bounding-box dimensions.

Framework	Model	AGAR (ms)	Curated (ms)
Detectron2	RetinaNet R-50	202	109
	RetinaNet R-101	237	142
	Faster R-CNN R-50	199	117
	Faster R-CNN R-101	237	146
	Mean	*219*	*128*
YOLOv8	YOLOv8s	22	17
	YOLOv8m	40	23
	YOLOv8l	64	41
	Mean	*42*	*27*

Table 2: Mean inference time (ms/image) by model and dataset.

Class	n	Median width	Median height	<32	<64	<128	<256	<512
S. aureus	13,966	47	47	14.3	82.0	100.0	100.0	100.0
P. aeruginosa	9,697	141	141	0.1	2.6	38.4	92.6	100.0
E. coli	14,024	162	166	0.0	0.6	24.2	91.8	100.0
Overall	37,687	112	114	5.3	31.3	56.0	95.0	100.0

Table 3: Anchor coverage analysis for the AGAR dataset. Columns <32 through <512 show the cumulative percentage (%) of annotations with bounding-box area below anchor^2. Median dimensions in pixels.

Class	n	Median width	Median height	<32	<64	<128	<256	<512
S. aureus	85	54	61	1.2	62.4	100.0	100.0	100.0
P. aeruginosa	106	61	65	0.0	53.8	93.4	100.0	100.0
E. coli	87	71	67	8.0	46.0	79.3	100.0	100.0
Overall	278	59	63	2.9	54.0	91.0	100.0	100.0

Table 4: Anchor coverage analysis for the curated dataset (test set). Same format as Table 3.

In the AGAR dataset, the anchor mismatch is class-specific: 82% of *S. aureus* annotations fall below the 64 px level, while *E. coli* and *P. aeruginosa* are well-covered by mid-range anchors. This explains the consistent difficulty of *S. aureus* detection across all Detectron2 configurations and the performance advantage of Faster R-CNN (which has a second-stage refinement) over RetinaNet for this class.

In the curated dataset, the objects are smaller overall (median 59x63 px overall), and the anchor mismatch affects all classes more uniformly than in AGAR. This likely contributes to the lower absolute mAP on the curated dataset. YOLOv8's anchor-free detection head bypasses this constraint, which may partly account for its stronger performance on smaller colonies.

4 Stress Test on Dense Plates

Images with more than 100 annotations per plate were excluded from the main training and evaluation sets to reduce noise from confluent growth. To quantify performance degradation on dense plates, all four Detectron2 models trained on the full AGAR dataset were evaluated on these excluded images, stratified by annotation density. Table 5 reports the results.

**Density**	**Model**	**mAP**	***S. aureus***	***P. aeruginosa***	***E. coli***
101–150	Faster R-CNN R-50	54.85	56.96	52.41	55.19
	Faster R-CNN R-101	54.69	56.13	52.28	55.65
	RetinaNet R-50	46.19	35.40	49.25	53.91
	RetinaNet R-101	46.23	35.79	49.08	53.82
151–300	Faster R-CNN R-50	45.17	36.38	47.65	51.49
	Faster R-CNN R-101	44.48	35.82	46.63	50.97
	RetinaNet R-50	33.92	12.08	43.32	46.38
	RetinaNet R-101	33.11	10.78	42.72	45.83
>100 (all)	Faster R-CNN R-50	49.85	46.23	50.84	52.49
	Faster R-CNN R-101	49.39	45.42	50.54	52.21
	RetinaNet R-50	38.99	21.43	47.37	48.17
	RetinaNet R-101	38.52	20.61	47.11	47.83

Table 5: Stress test: Detectron2 model performance (%) on dense plates excluded from the main evaluation (images with >100 annotations). Performance declines with increasing density, with RetinaNet and *S. aureus* detection most affected.

Performance declines predictably with plate density. Faster R-CNN R-50 drops from 62.6% mAP on the main test set to 54.9% at 101–150 annotations and 45.2% at 151–300. RetinaNet shows a sharper decline, particularly for *S. aureus* (from 37.3% to 12.1% at the highest density tier). The two-stage architecture of Faster R-CNN provides a more graceful degradation under crowded conditions.

5 Training Budget Ablation

The Detectron2 models were trained for 10 epochs and the YOLOv8 models for 100 epochs. To assess whether this difference confounded the architectural comparison, Faster R-CNN R-101 was additionally trained on the full AGAR dataset with a maximum of 100 epochs. The best validation AP was reached at iteration 16,235, corresponding to approximately epoch 22 (738 iterations per epoch). Evaluation of the best checkpoint on the test set yielded 62.5% mAP, comparable to the original 10-epoch result (63.1%). This indicates that extending Detectron2 training beyond the original budget did not materially improve the best Faster R-CNN R-101 result, suggesting that training duration alone does not account for the gap relative to YOLOv8.
